# Supplementary material for: Could Ovarian Cancer Prediction Models Improve the Triage of Symptomatic Women in Primary Care? A Modelling Study Using Routinely Collected Data
Source: Cancers (Basel). 2021 Jun 9;13(12):2886. doi: 10.3390/cancers13122886 (PMC8228892; doi:10.3390/cancers13122886)
Supplement: Supplementary file 1 [file cancers-13-02886-s001.zip › cancers-1229294-supplementary.pdf]

cancers MDPI

Supplementary material: Could Ovarian Cancer Prediction Models Improve the Triage of Symptomatic Women in Primary Care? A Modelling Study Using Routinely Collected Data

Garth Funston, Gary Abel, Emma J. Crosbie, Willie Hamilton and Fiona M. Walter

Table S1. Completed TRIPOD checklist.

Section/Topic	Item	Checklist Item	location
		**Title and abstract**	
Title	1	Identify the study as developing and/or validating a multivariable prediction model, the target population, and the outcome to be predicted.	Title
Abstract	2	Provide a summary of objectives, study design, setting, participants, sample size, predictors, outcome, statistical analysis, results, and conclusions.	Abstract
		**Introduction**	
Background and objectives	3a	Explain the medical context (including whether diagnostic or prognostic) and rationale for developing or validating the multivariable prediction model, including references to existing models.	Introduction para 1-3
	3b	Specify the objectives, including whether the study describes the development or validation of the model or both.	Introduction para 4
		**Methods**	
Source of data	4a	Describe the study design or source of data (e.g., randomized trial, cohort, or registry data), separately for the development and validation data sets, if applicable.	Methods para 2
	4b	Specify the key study dates, including start of accrual; end of accrual; and, if applicable, end of follow-up.	Methods para 3
Participants	5a	Specify key elements of the study setting (e.g., primary care, secondary care, general population) including number and location of centres.	Methods para 3
	5b	Describe eligibility criteria for participants.	Methods para 3
	5c	Give details of treatments received, if relevant.	N/A
Outcome	6a	Clearly define the outcome that is predicted by the prediction model, including how and when assessed.	Methods para 4
	6b	Report any actions to blind assessment of the outcome to be predicted.	N/A
Predictors	7a	Clearly define all predictors used in developing or validating the multivariable prediction model, including how and when they were measured.	Methods para 5 and table 1
	7b	Report any actions to blind assessment of predictors for the outcome and other predictors.	N/A
Sample size	8	Explain how the study size was arrived at.	Methods para 3 and 9
Missing data	9	Describe how missing data were handled (e.g., complete-case analysis, single imputation, multiple imputation) with details of any imputation method.	Methods para 9
Statistical analysis methods	10a	Describe how predictors were handled in the analyses.	Methods para 5, 7,8 table 1 S1 Text
	10b	Specify type of model, all model-building procedures (including any predictor selection), and method for internal validation.	Methods para 9
	10d	Specify all measures used to assess model performance and, if relevant, to compare multiple models.	Methods para 10 and 11

Risk groups	11	Provide details on how risk groups were created, if done.	Methods para 11
		**Results**	
Partici-pants	13a	Describe the flow of participants through the study, including the number of participants with and without the outcome and, if applicable, a summary of the follow-up time. A diagram may be helpful.	Results para 1 and Figure 1
	13b	Describe the characteristics of the participants (basic demographics, clinical features, available predictors), including the number of participants with missing data for predictors and outcome.	Results para 2 and Table 2
Model de-velopment	14a	Specify the number of participants and outcome events in each analysis.	N/A
	14b	If done, report the unadjusted association between each candidate predictor and outcome.	N/A
Model specifica-tion	15a	Present the full prediction model to allow predictions for individuals (i.e., all regression coefficients, and model intercept or baseline survival at a given time point).	S2 Table
	15b	Explain how to the use the prediction model.	Results para 5-6
Model per-formance	16	Report performance measures (with CIs) for the prediction model.	Para 4-6 and Tables 2 and 3
		**Discussion**	
Limita-tions	18	Discuss any limitations of the study (such as nonrepresentative sample, few events per predictor, missing data).	Discussion Para 2-5
Interpreta-tion	19b	Give an overall interpretation of the results, considering objectives, limitations, and results from similar studies, and other relevant evidence.	Discussion para 1, 6 and 8
Implica-tions	20	Discuss the potential clinical use of the model and implications for future research.	Discussion para 7-11
		**Other information**	
Supple-mentary infor-mation	21	Provide information about the availability of supplementary resources, such as study protocol, Web calculator, and data sets.	"Institut. review board" and "Data availability" sections
Funding	22	Give the source of funding and the role of the funders for the present study.	"Funding" section

Text S1. Preparation of candidate variables.

Age

Patient age in years on the date of CA125 testing was determined from CPRD records.

Ethnicity

Ethnicity is recorded within the CPRD using hierarchical codes which map to 1991 and 2001 census categories. A Read code list, developed by Mathur *et al*, was used to identify codes for ethnicity recorded at any point within a woman's CPRD record [1]. These codes fall into 5 overarching groups: White, Mixed, Asian, Black and Other ethnicities [2]. A patient may have codes for different ethnicities recoded within the CPRD. We followed the approach taken by Mathur *et al* to determine patient ethnicity from CPRD [2]:

1. If a single ethnic group was recoded we accept that group
2. If more than one ethnic group was recorded we accepted the most common ethnic group
3. If more than one ethnic group was recorded with the same frequency (i.e. a 'tie') we accepted the most recent ethnic group recorded
4. If there was a tie on the most recent date, CPRD ethnicity treated as missing

Not all patients have an ethnicity recorded within CPRD. Where ethnicity data was missing in CPRD, we used the HES APC ethnicity code.

Ethnicity was initially categorised into the five groups, but numbers of women in individual ethnic groups, other than White, were small e.g. 160 women (0.56%) were recorded as Mixed ethnicity. So, ethnicity was further collapsed into two groups: "White" and "other ethnicities". Multiple imputation was used to replace missing ethnicity where none could be identified either from the CPRD or HES APC files.

Height

Patient height was determined from CPRD records. Heights recorded in metres were converted to cm. Heights recorded during childhood (<18 years) and implausible values (<121cm and >214cm) were excluded [3]. The most recent height, recorded on or prior to the CA125 test date, was identified for each woman. Multiple imputation was used to replace missing height data.

Body Mass Index (BMI)

BMI was calculated for each woman using: a) the most recent plausible (>20kg) adult (≥18 years) weight recorded in the CPRD in the ten years prior to the CA125 test date, and b) the most recent height (excluding <1.21m and >2.14m) recorded on or prior to the CA125 test date. Plausible ranges were informed by the literature [3,4]. Where more than one weight or height was recorded on the same day, the mean was used in the BMI calculation. As in other CPRD studies [3,5], where the BMI could not be calculated directly from weight and height due to missing data, the most recent directly entered BMI value (recorded in the ten years prior to the CA125 test date) was accepted. Implausible BMI measurements (<5kg/m^2 and >200kg/m^2) were excluded [3,5]. Multiple imputation was used to replace missing BMI data.

Personal history of breast cancer

In situ or invasive primary breast cancers recorded in either the NCRAS (ICD10 codes C50 and D05) or CPRD (relevant Read codes) on or prior to the CA125 test date were identified. Women were classified as either having or not having a personal history of breast cancer.

Symptoms

A Read code list was used to identify women with symptoms of ovarian cancer coded within the CPRD in the year before CA125 testing. The symptoms chosen were those listed

in current NICE guidelines on ovarian cancer detection in primary care [6]. Each of the nine symptoms were classified as either present or absent for each patient.

CA125

Preparation of CA125 is described in the "Participants" section in the main text of this paper.

Platelet count

Platelet counts, recorded in the test file of CPRD on or in the 12 months preceding the CA125 test date, were identified. Where multiple platelet counts existed, the most recent was used. Where multiple platelet counts occurred on the same day, the mean was taken.

The standard upper reference range for platelets is 450 x 10^9/L. However, there evidence that patients with 'high normal' platelet counts in primary care have a greater risk of cancer than those with 'low normal' counts. This was taken into account when categorising platelets [7]. Four categories were used:

1. Not tested
2. <300 × 10^9/L
3. 300–449×10^9/L
4. ≥450 × 10^9/L

Haemoglobin level

Haemoglobin levels, recorded in the test file of CPRD on or in the 12 months preceding the index test date, were identified. Entries recorded in g/L where converted to g/dl. Where multiple haemoglobin levels existed, the most recent was used. Where multiple haemoglobin levels were recorded on the same day, the mean was taken.

Three categories were used:

1. Not tested
2. <12g/dl
3. ≥12g/dl

Albumin level

Albumin levels, recorded in the test file of CPRD on or in the 12 months preceding the CA125 test date, were identified. Where multiple levels existed, the most recent was used. Where multiple albumin levels were recorded on the same day, the mean was taken.

Three categories were used:

1. Not tested
2. <35 g/L
3. >35 g/L

CRP level

CRP levels, recorded in the test file of CPRD on or in the 12 months preceding the CA125 test date, were identified. Where multiple levels were recorded, the most recent was used. Where multiple CRP levels were recorded on the same day, the mean was taken. Four categories were used:

1. Not tested
2. <3 mg/L
3. 3–9.99 mg/L
4. ≥10 mg/L

Table S2. Model specifications.

Variable	Model 1 Coef. (95% CI)	Model 1 Odds ratio (95% CI)	Model 2 Coef. (95% CI)	Model 2 Odds ratio (95% CI)
**Baseline risk factors**				
Age: Age spline 1	−0.079 (−0.136 – -0.022)	0.924 (0.873 – 0.978)	−0.077 (−0.136 – -0.018)	0.926 (0.873 – 0.982)
Age spline 2	0.537 (0.223 – 0.852)	1.712 (1.250 – 2.345)	0.520 (0.199 – 0.841)	1.682 (1.220 – 2.319)
Age spline 3	−2.169 (−3.910 – -0.428)	0.114 (0.020 – 0.652)	−2.025 (−3.798 – -0.253)	0.132 (0.022 – 0.777)
Age spline 4	1.712 (−0.516 – 3.940)	5.539 (0.597 – 51.420)	1.542 (−0.724 – 3.808)	4.674 ($2.81x10^{-6}$ – 0.018)
Ethnicity: White			Reference	Reference
Other			−0.906 (−1.756 – -0.055)	0.404 (0.173 – 0.947)
Log BMI			0.965 (0.224 – 1.705)	2.624 (1.251 – 5.503)
Height (cm)			0.040 (0.017 – 0.062)	1.041 (1.017 – 1.064)
**Symptoms**				
Abdominal / pelvic pain			0.412 (0.089 – 0.735)	1.510 (1.093 – 2.087)
Distension			0.648 (0.034 – 1.261)	1.911 (1.035 – 3.530)
**Tests**				
Log CA125: Log CA125 spline 1	1.129 (−2.386 – 4.643)	3.092 (0.092 – 103.862)	1.043 (−2.429 – 4.516)	2.839 (0.088 – 91.447)
Log CA125 spline 2	-7.114 (−26.469 – 12.241)	0.0008 ($3.19x10^{-12}$ – $2.07x10^{5}$)	-6.592 (−25.805 – 12.622)	0.001 ($6.21x10^{-12}$ – $3.03x10^{5}$)
Log CA125 spline 3	82.537	$7.01x10^{35}$	78.551	$1.30x10^{34}$

	(−17.899 – 182.973)	($1.69x10^{-08}$ – $2.91x10^{79}$)	(−21.420 – 178.521)	($4.98x10^{-10}$ – $3.39x10^{77}$)
Log CA125 spline 4	-143.749<br>(−267.564 – -19.934)	$3.72x10^{-63}$<br>($6.3x10^{-117}$ – $2.20x10^{-09}$)	-137.307<br>(−260.776 – -13.839)	$2.33x10^{-60}$<br>($5.6x10^{-114}$ – $9.77x10^{-07}$)
Platelets:<br>No test			Reference	Reference
<$300x10^9$/L			−0.699<br>(−1.350 – -0.048)	0.497<br>(0.259 – 0.953)
300 – 449 $x10^9$/L			−0.378<br>(−1.053 – 0.297)	0.685<br>(0.349 – 1.346)
≥450 $x10^9$/L			−0.103<br>(−0.885 – 0.678)	0.902<br>(0.413 – 1.971)
Albumin:<br>No test			Reference	Reference
<35 g/L			−1.241<br>(−1.951 – -0.531)	0.289<br>(0.142 – 0.588)
≥35 g/L			−0.106<br>(−0.625 – 0.413)	0.899<br>(0.535 – 1.511)

Coef. = variable coefficient. CI = confidence interval

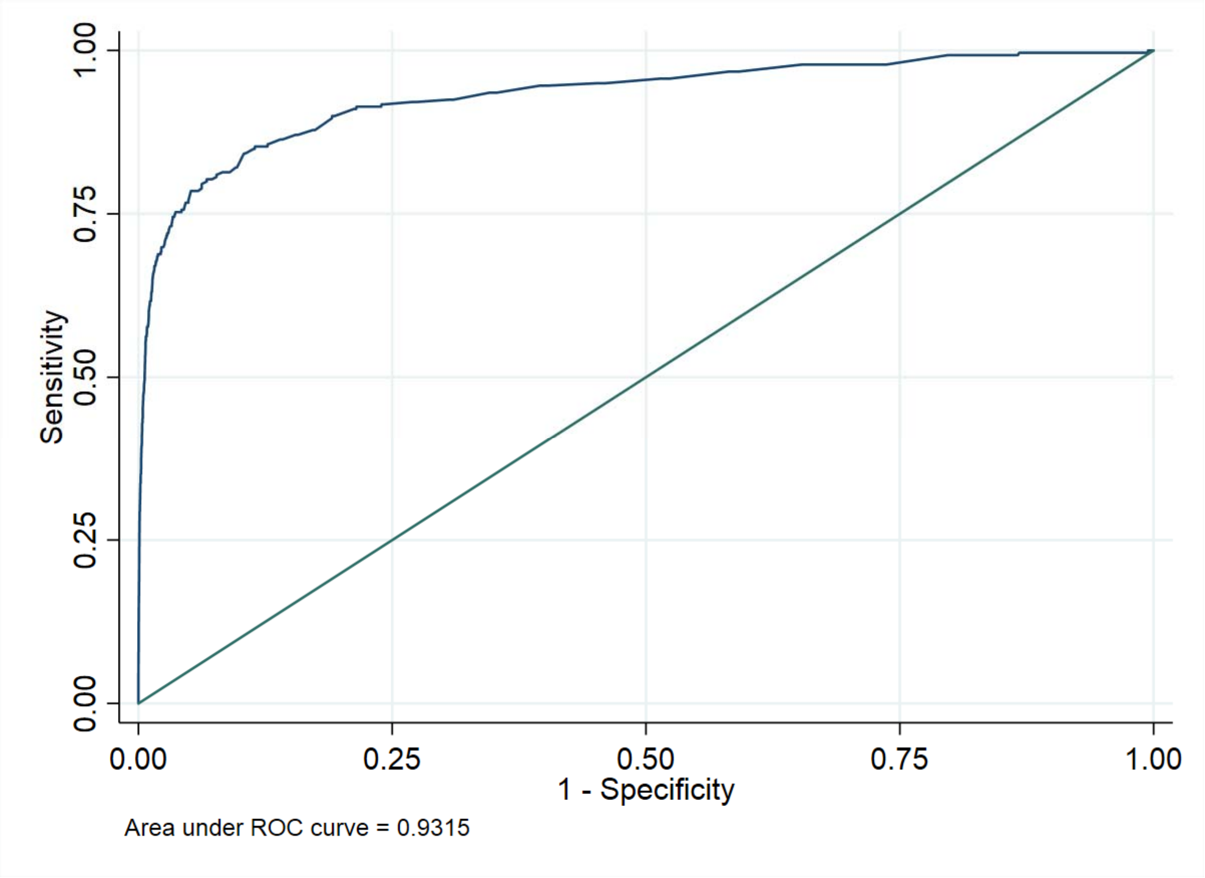

Figure S1. ROC curve for CA125.

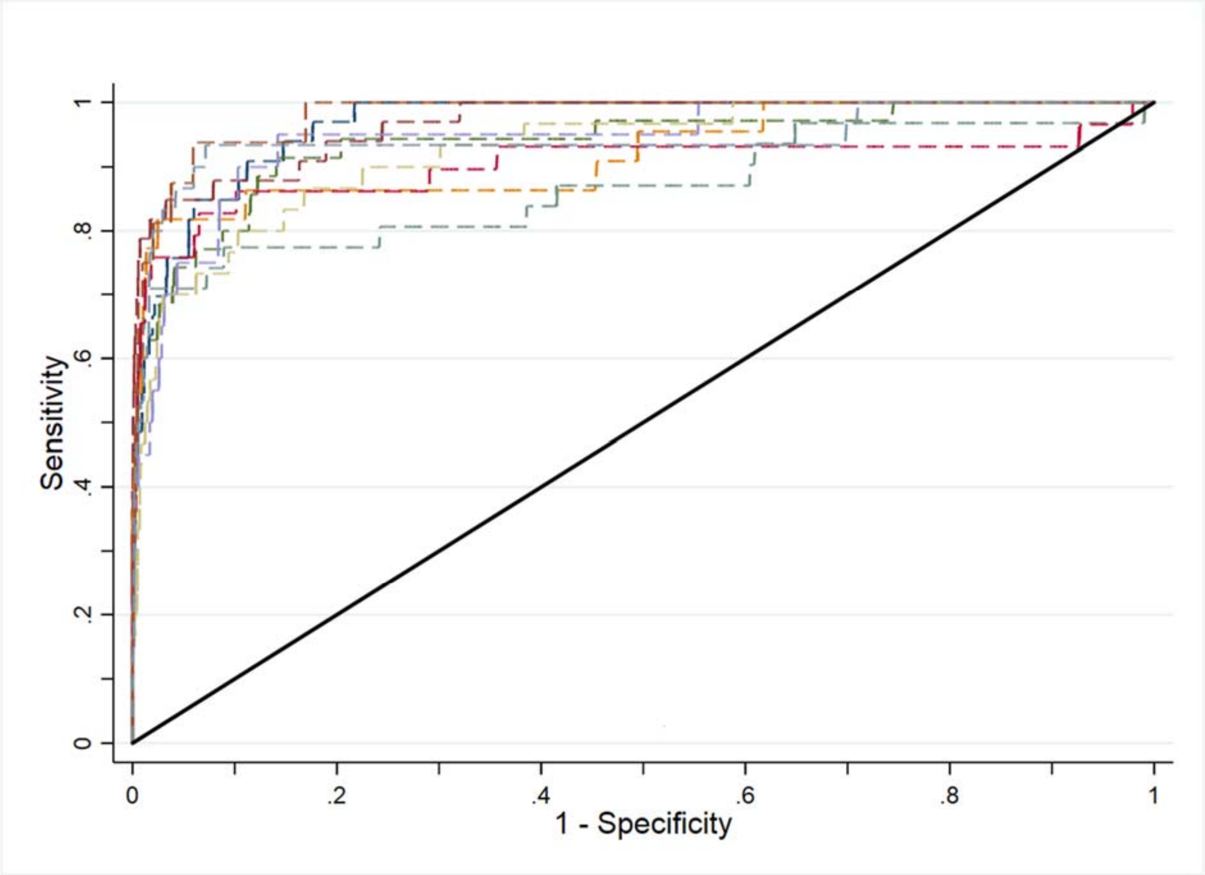

Figure S2. Tenfold cross-validation ROC curve for Model 1.

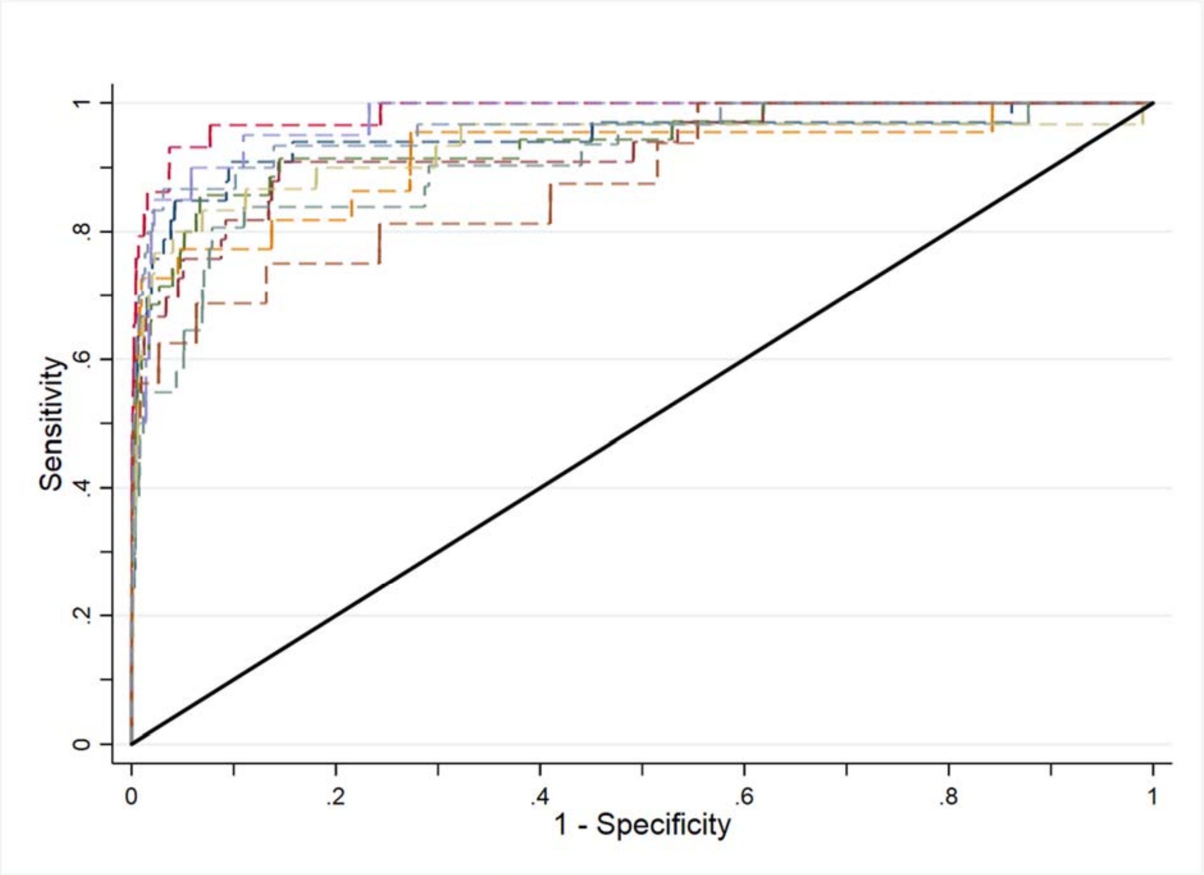

Figure S3. Tenfold cross-validation ROC curve for Model 2.

This ROC curve was prepared using imputation set 20 as an example. To calculate the overall cross-validation AUC for Model 2, the cross-validation AUC was calculated for each of the 20 imputed datasets and Rubin's rules were used to combine results across the imputed datasets.

References

1 Mathur R, Palla L, Farmer RE, Chaturvedi N, Smeeth L. Ethnic differences in the severity and clinical management of type 2 diabetes at time of diagnosis: A cohort study in the UK Clinical Practice Research Datalink. *Diabetes. Res. Clin. Pract.* **2020**, *160*, 108006.

2. Mathur, R.; Bhaskaran, K.; Chaturvedi, N.; Leon, D.A.; VanStaa, T.; Grundy, E.; Smeeth, L. Completeness and usability of ethnicity data in UK-based primary care and hospital databases. *J. Public Health* **2014**, *36*, 684–692.

3. Bhaskaran K, Forbes HJ, Douglas I, Leon DA, Smeeth L. Representativeness and optimal use of body mass index (BMI) in the UK Clinical Practice Research Datalink (CPRD). *BMJ. Open.* **2013**, *3*, e003389.

4. Nicholson BD, Aveyard P, Hamilton W, Bankhead CR, Koshiaris C, Stevens S, Hobbs FDR, Perera R. The internal validation of weight and weight change coding using weight measurement data within the UK primary care electronic health record. *Clin. Epidemiol.* **2019**, *11*, 145–55.

5. Bhaskaran K, Douglas I, Forbes H, Dos-Santos-Silva I, Leon DA, Smeeth L. Body-mass index and risk of 22 specific cancers: A population-based cohort study of 5·24 million UK adults. *Lancet* 2014, **384**, 755–65.

6. National Institute for Health and Care Excellence. *Suspected Cancer: Recognition and Referral (NG12)*; NICE: London, UK, 2015.

7. Mounce LTA, Hamilton W, Bailey SER. Cancer incidence following a high-normal platelet count: cohort study using electronic healthcare records from English primary care. *Br. J. Gen. Pract.* **2020**, *70*, e622-e628.
